# Spatial analysis for bovine viral diarrhea virus and bovine herpesvirus type 1 infections in the state of Paraíba, northeastern Brazil

**DOI:** 10.1186/s12917-018-1412-5

**Published:** 2018-03-20

**Authors:** Leíse Gomes Fernandes, Edviges Maristela Pituco, Adriana Hellmeister de Campos Nogueira Romaldini, Eliana De Stefano, Inácio José Clementino, Amanda Rafaela Alves Maia, Carolina de Sousa Américo Batista Santos, Clebert José Alves, Sérgio Santos de Azevedo

**Affiliations:** 1 Laboratory of Transmissible Diseases, Academic Unit of Veterinary Medicine, Center of Rural Technology and Health, Federal University of Campina Grande, Patos, PB 58700-970 Brazil; 2 Laboratory of Bovidae Viruses, Biological Institute, São Paulo, SP 04014-900 Brazil; 3 Department of Veterinary Medicine, Federal University of Paraíba, Areia, PB 58397-000 Brazil

**Keywords:** Cattle, Epidemiology, Cluster analysis, BVDV, BoHV-1, Kriging estimate

## Abstract

**Background:**

Bovine Viral Diarrhea Virus (BVDV) and Bovine Herpesvirus type 1 (BoHV-1) cause reproductive problems in cattle and restrictions on international trade in animals worldwide. Both infections were detected in cattle herds in the Paraíba state, Northeastern Brazil, however, the spatial distribution and geographic identification of positive herds for these viruses has never been examined. Therefore, the aim of this study was to describe the spatial pattern of apparent prevalence estimate and to identify spatial clustering of positive herds of BVDV and BoHV-1 infections in cattle herds from the state of Paraíba, Northeastern Brazil.

**Results:**

The herd-level prevalence for BVDV and BoHV-1 infections in Paraíba were, respectively, 65.5% (95% CI: 61.1**–**69.7) and 87.8% (95% CI: 84.5**–**90.5). The average apparent within-herd prevalence of BVDV was 31.8% and of BoHV-1 was 62.4%. The predicted prevalence was highest (0.42–0.75) for BVDV in the west, north and eastern part of Sertão and in the central and eastern part of Agreste/Zona da Mata. For BoHV-1, the highest predicted prevalence (0.74–0.97) was in some local areas across Sertão and throughout the eastern part of Agreste/Zona da Mata. Six significant clusters were detected for BVDV, a primary cluster covering the eastern Sertão region, with 11 herds, radius of 24.10 km and risk relative (RR) of 2.21 (*P* <  0.001) and five smaller significant clusters, involving one or two herds in Agreste/Zona da Mata region with a high RR. A significant clustering of BoHV-1 positive herds (*P* <  0.001) was detected in Agreste/Zona da Mata region with a radius of 77.17 km and a RR of 1.27, with 103 cases. Consistency was found between kriging and SatScan results for identification of risk areas for BVDV and BoHV-1 infections.

**Conclusions:**

The clusters detected contemplated different areas of the state, with BVDV cluster located in the Sertão and BoHV-1 in Agreste/Zona da Mata stratum. Through the risk mapping, it was possible to identify the areas in which the risk is significantly elevated, coincided with areas where there are borders with other states and in which there is a high movement of animals.

## Background

The reproductive infectious diseases are those that cause more damage in productivity in cattle herds, assuming importance viral agents, especially Bovine Viral Diarrhea Virus (BVDV) and Bovine Herpesvirus type 1 (BoHV-1), which cause reproductive problems in both beef and milk cattle, and restrictions on international trade for animals and animal products [1–3].

BVDV is a member of the genus *Pestivirus,* which belongs to the *Flaviviridae* family, and although capable of manifesting in different clinical presentations and acute, sub acute or chronic character, the reproductive consequences of this virus and its persistent infection have greater economic and epidemiological importance [4, 5]. The transmission may occur by a wide range of body fluids (nasal discharge, urine, milk, semen, saliva and fetal fluids), indirect contacts (airborne transmissions, contaminated material or iatrogenic) and via the placenta to the fetus, resulting in the birth of an immunotolerant and persistently infected (PI) calf [6]. The PI animals result from uterine exposure to noncytopathic strains of BVDV before development of fetal immunocompetence (generally before 125 days of gestation) [7]. PI calves are often weak at birth or die before one year of age, but others may not show clinical signs, being epidemiologically important due to shed large amounts of virus, acting as constant sources of infection for susceptible animals [8]. The detection and removal of PI animals and vaccination of susceptible animals are key strategies for the control of herds from BVDV [9, 10].

The BoHV-1 is a alphaherpesvirus belonging to the genus *Varicellovirus*, which infects cattle and presents clinical manifestations such as pustular vulvovaginitis or balanoposthitis, abortion, rhinotracheitis and meningoencephalitis, and causes great economic losses to the livestock industry [11, 12]. This virus may be transmitted by respiratory and genital secretions of infected individuals through direct and indirect contact, such as contaminated materials, contaminated semen or airbone transmission [13]. BoHV-1 has the ability to cause latent infections, and periodically, BoHV-1 may be reactivated, and then shed and transmitted, which guarantees its perpetuation and spread in herds, thus being the main obstacle to the establishment of control measures [14].

The seroprevalence of BVDV and BoHV-1 have numerous references available all over the world, with variable values, but generally higher, depending on the control measures practiced [15]. In Brazil, regional data obtained from serological surveys reveal significant spread of the virus in beef and dairy herds [16–18]. In the state of Paraíba, Thompson et al. [19] found anti-BVDV antibodies in 22.2% of animals and 88.9% of herds and anti-BoHV-1 antibodies in 46.6% of animals and 100% of herds. These authors concluded that the within-herd spread of BVDV and BoHV-1 were relatively slow and its ubiquitous nature makes it difficult to generalize the rate of among-herds spread.

BVDV and BoHV-1 infections occur in cattle herds in the state of Paraíba, however, the spatial distribution and geographic identification of positive herds for these viruses has never been examined. According to Carpenter [20], whether it is an outbreak investigation or epidemiological research, more emphasis should be placed on the spatial and temporal components of health events in order to identify unusual occurrences of events that happen close together in either time and/or space.

Spatial analysis of infectious diseases allows for the detection of disease clusters, which can occur due to common risk factors among herds or the transmission between neighbors herds, being a useful tool in epidemiological surveillance providing better visualization and hypothesis survey for the occurrence of clusters, facilitating the elaboration of control strategies [21]. Hence, the present study aimed to describe the spatial pattern of apparent prevalence estimate of BVDV and BoHV-1 infections in cattle herds from the state of Paraíba, Northeastern Brazil, including identification of areas with increased risk of occurrence of these viruses.

## Methods

### Characterization of the study area

The state of Paraíba, located in the Northeastern region of Brazil, is characterized by warm weather throughout the year. The state is geographically subdivided into the following four regions, based mostly on vegetation type and rainfall: (i) Zona da Mata (Atlantic forest), (ii) Agreste, (iii) Borborema, and (iv) Sertão. The Zona da Mata and Agreste have relatively higher rainfall regimes [22]. Borborema and Sertão (semi-arid region) are typically within the Caatinga biome, a xeric forest and a forest of thorns composed by cacti, thick plants, spiny brush and adapted arid grasses [23]. The climate is characterized by a warm and semi-arid climate, with average temperatures of 27 °C, and the average annual rainfall is typically ≈500 mm. There are typically two seasons: a rainy season from February to May, and a long drought period from June to January. However, occurrences of droughts lasting more than one year are also characteristic of the region [24].

The animal husbandry has an increasingly important in the Agreste, Borborema and Sertão regions, where small cattle-raising farms and family farms are widespread. The cattle are usually reared extensively on native Caatinga in most of the Borborema and Sertão farms. Following the Brazilian scenario of milk production, in the state of Paraíba around 69% of milk was produced in small cattle-raising farms [25].

### Data source

The data used in this study were obtained from a survey of bovine brucellosis in the state of Paraíba made by the National Program for Control and Eradication of Brucellosis and Tuberculosis, in which samples were collected from September 2012 to January 2013. In total, 2443 cows aged ≥24 months were sampled from 478 herds.

### Study design

**Fig. 1:**
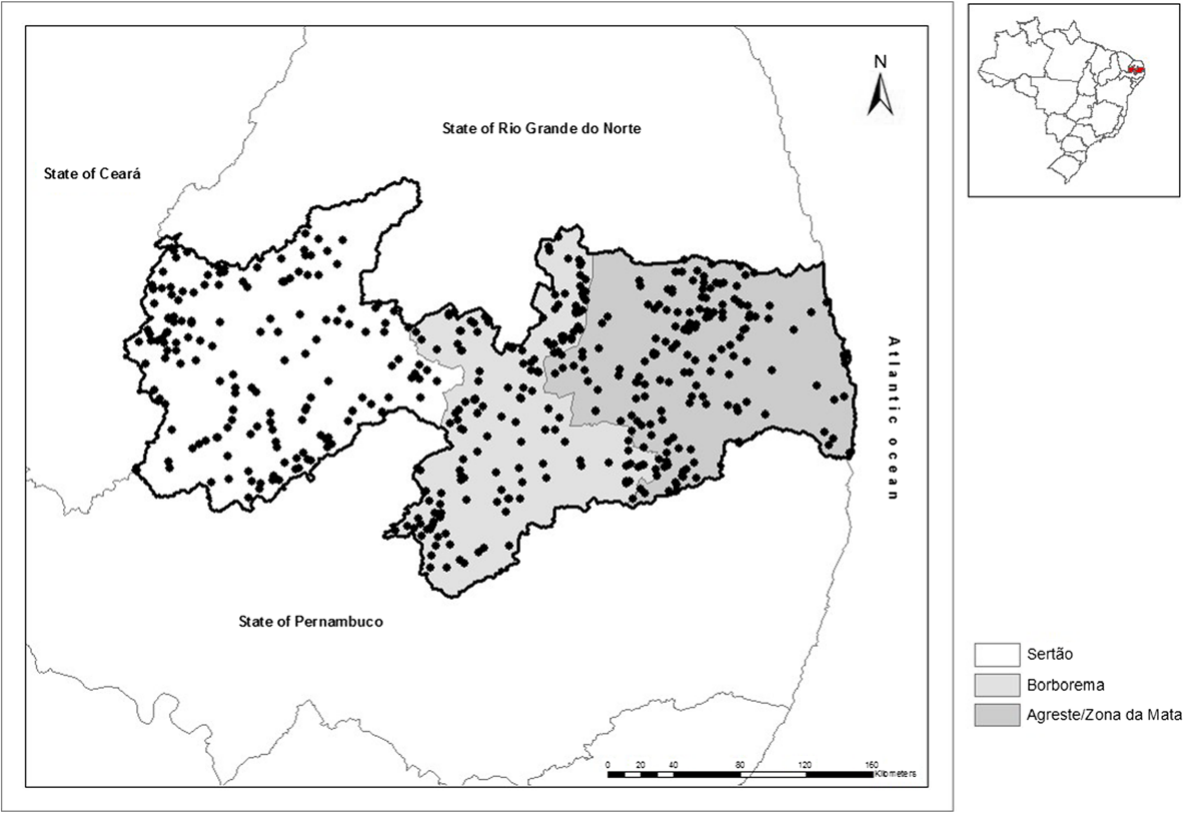
Spatial localization of cattle herds in the State of Paraíba, Northeastern Brazil, according to sampling stratum. Detail shows Paraíba state within Brazil

The state of Paraíba was divided into three sampling groups: sampling stratum 1 (mesoregion of Sertão), sampling stratum 2 (mesoregion of Borborema), and sampling stratum 3 (mesoregions of Zona da Mata and Agreste) (Fig. 1). When this stratification scheme was proposed, the operational capacity of the Agricultural and Livestock Defense Service of the State of Paraíba (SEDAP) was considered based on the areas of action of its regional units in order to ensure that the veterinarians and agricultural and livestock technicians from the SEDAP could conduct the fieldwork. For each sampling stratum, a pre-established number of herds with reproductive activity (primary sampling units) were randomly selected and then, a pre-established number of unvaccinated cows aged ≥24 months were randomly and systematic selected (secondary sampling units), using the following criteria: 10 animals were sampled in herds with up to 99 cows aged over 24 months; 15 animals were sampled in herds with 100 or more cows aged over 24 months; and all animals were sampled in those with up to 10 cows aged over 24 months.

The field activities included blood collection and sending the samples to the laboratory. The coordinates in each herd were identified using a global positioning system device (GPS).

### BoHV-1 and BVDV serological diagnosis

For the serological diagnosis of BVDV and BoHV-1 infections the virus-neutralization test was used [26, 27]. The cytopathic viral strains BVDV-1 (NADL) and BoHV-1 supplied by the Virology Institute of the Veterinary Medicine College of Hanover, Germany were used. The technique was performed in two stages, screening and titration, and a sample was considered positive when it presented a titer ≥10 for BVDV and ≥2 for BoHV-1. The neutralizing antibodies titers were considered the reciprocal of the higher serum dilutions capable of inhibiting the viral replication and the consequent production of cytopathic effect of BVDV and BoHV-1. The infectious titer (TCID_50:_ infective doses for 50% of cell cultures) used was 10^5.61^TCID_50_/50 μL for BVDV and 10^6.25^ TCID_50_/50 μL for BoHV-1, determined by the Reed and Müench method [28].

### Herd-level case definition

The herd-level case definition was based on the size of the population (cows aged ≥24 months), number of females sampled, an intra-herd apparent prevalence of 50%, and the sensitivity and specificity of the virus-neutralization test of 95 and 99.5% for BVDV [29] and 94.4% and 93.2% for BoHV-1 [30], respectively, with the goal of obtaining a herd sensitivity and specificity of ≥90%. After simulations using Herdacc software, a herd was deemed positive for BVDV when at least one positive animal was detected, and for BoHV-1 infection, a herd was considered positive if it included at least one positive animal in herds of up to seven females; two positive animals in herds of 8–99 females; and three positive animals in herds with more than 99 females.

### Apparent prevalence calculations

EpiInfo 6.04 software was used to calculate the apparent prevalences and respective confidence intervals [31]. Stratified random sampling was used to calculate the herd-level prevalence in the state of Paraíba [32]. The required parameters were as follows: (a) condition of the herd (positive or negative), (b) sampling stratum to which the herd belonged, and (c) statistical weight. The statistical weight was determined by applying the following formula [31]:

$$ Weight=\frac{\mathrm{number}\ \mathrm{of}\ \mathrm{herds}\ \mathrm{in}\ \mathrm{the}\ \mathrm{stratum}\kern1em }{\mathrm{number}\ \mathrm{of}\ \mathrm{herds}\ \mathrm{sampled}\ \mathrm{in}\ \mathrm{the}\ \mathrm{stratum}} $$

The calculation of the herd-prevalence per sampling stratum employed the sampling design of a simple random sample by using the following parameters: (a) number of positive herds and (b) number of herds sampled in the stratum.

### Spatial analysis

Identification of the herd, geographical coordinates and results of serological tests were included in a database for spatial analysis. Firstly, the Cuzick–Edwards’ k-nearest neighbor method [33] was used to detect the possibility of spatial clustering at herd level using the ClusterSeer 2.5.1 software (BioMedware, Ann Arbor, MI, United States). Existence of potential spatial clustering was analyzed at each of the first 10 neighborhood levels, and the overall *p*-value was adjusted for multiple comparisons with the Simes approach. In a second step, scan statistics by the SatScan software version 9.0 [34] was used to identify local clusters of positive herds. A Bernoulli model was applied, the scanning window was circular, and the spatial size of scan window was limited to 25% of the total population. Because of the high herd-level prevalences for BVDV and BoHV-1, analysis was not run on herd-level, and then considering within-herd prevalence. The statistical significance level was set as 0.05 and the maps were constructed with the ArcGIS software.

The kriging method was used to estimate the spatial pattern of apparent within-herd prevalence of BoHV-1 and BVDV. By this method, it is possible to produce a smooth surface of predicted prevalence and a map of prediction variance [35]. Kriging is based on the spatial correlation between measurements which is modelled by the semivariogram, and work well on stationary and non-stationary rates [36]. Directional semivariograms in four directions (north, north-east, east, south-east) were fitted to test for anisotropy. It was used an exponential model described below and fitted to the sample semivariogram [35]:

$$ \upgamma (d)={C}_o+C\left(1-\exp \left(-\frac{d}{a}\right)\right) $$

where the parameters were nugget (*c*_*o*_), partial sill (*c*), and range (*a*).

## Results

**Fig. 2:**
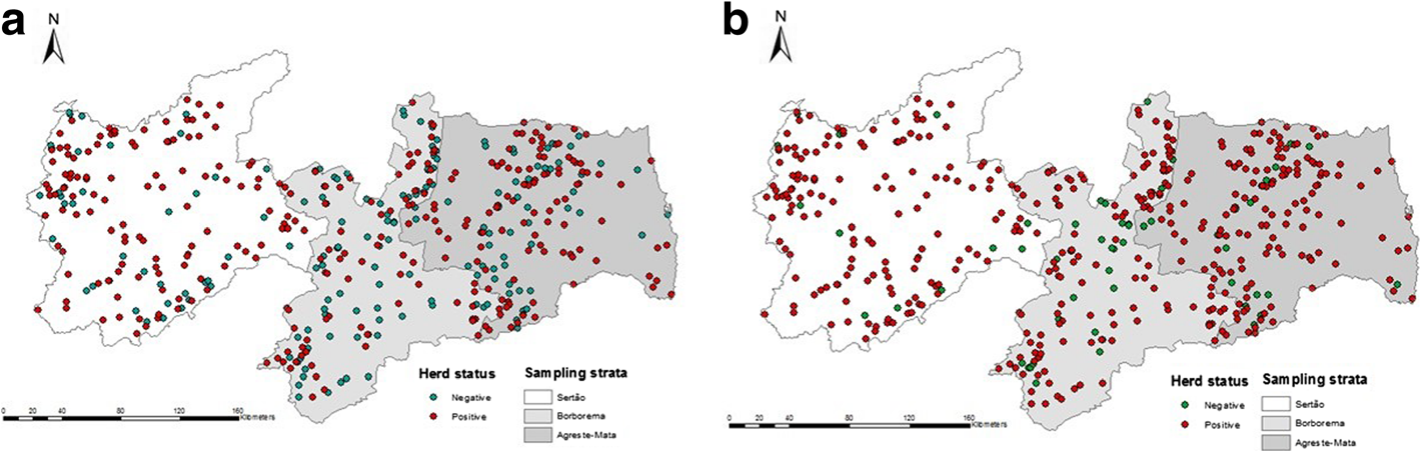
Spatial localization of positive and negative herds in the State of Paraíba, Northeastern Brazil: **a** Herd status of BVDV; **b** Herd status of BoHV-1

**Fig. 3:**
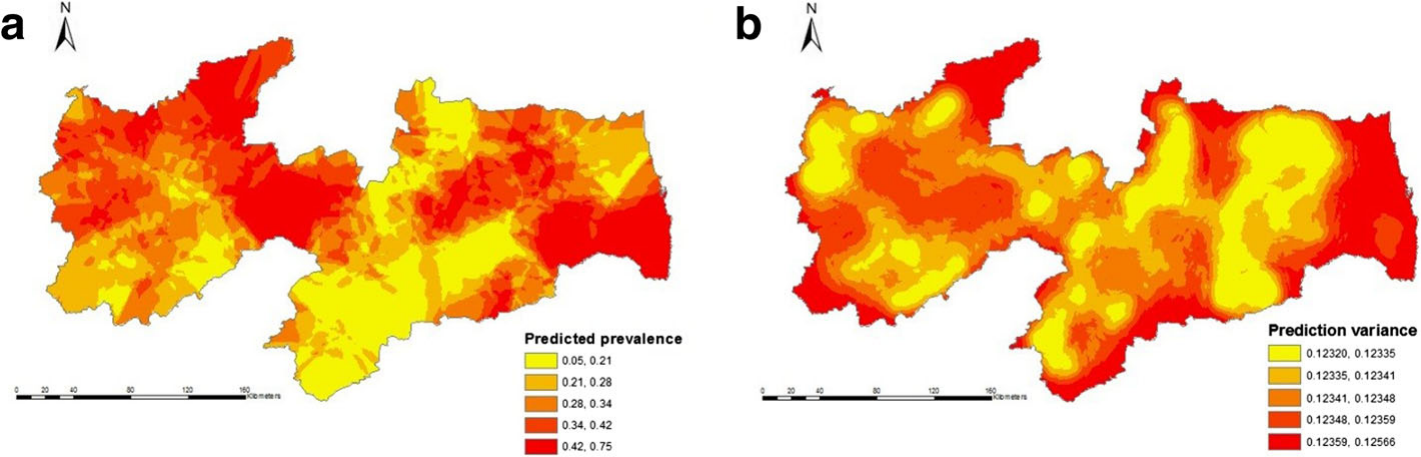
Apparent within-herd prevalences for BVDV in the State of Paraíba, Northeastern Brazil: **a**) Kriging surface of predicted apparent prevalence and (**b**) the variance of kriging estimates of predicted apparent prevalence

**Fig. 4:**
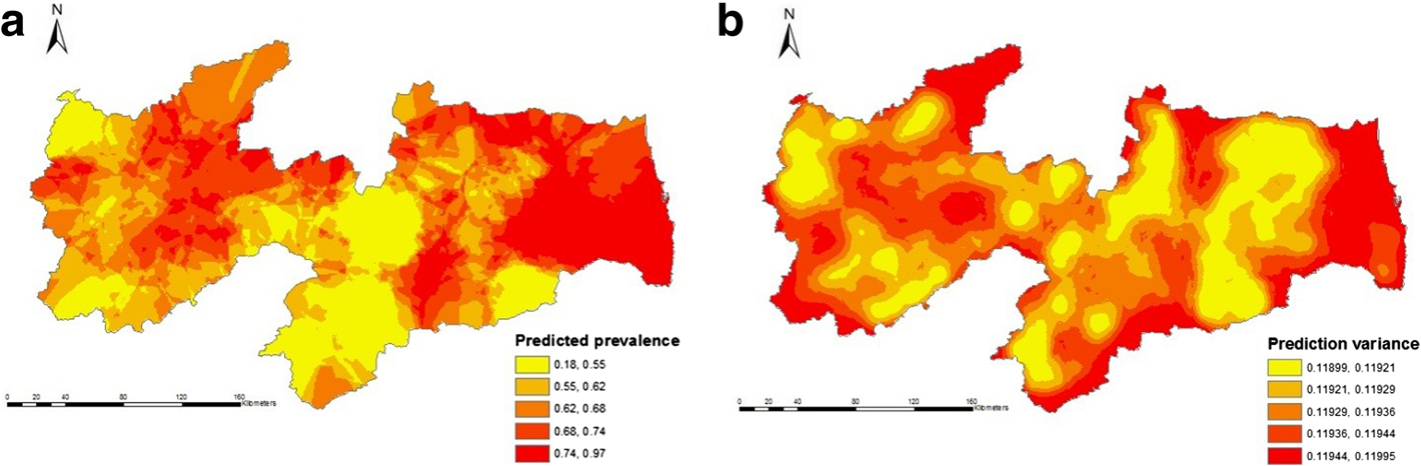
Apparent within-herd prevalences for BoHV-1 in the State of Paraíba, Northeastern Brazil: **a** Kriging surface of predicted apparent prevalence and (**b**) the variance of kriging estimates of predicted apparent prevalence

Figure 1 shows the spatial location of the herds in the state of Paraíba. The herd-level prevalence for BVDV and BoHV-1 infections in Paraíba were, respectively, 65.5% (95% CI: 61.1–69.7) and 87.8% (95% CI: 84.5–90.5) (Table 1). Two proprieties were excluded because they showed errors in their geographic coordinates, totaling 476 herds used in the spatial analysis. Figure 2 shows the spatial localization of positive and negative herds for BVDV (Fig. 2a) and BoHV-1 (Fig. 2b). The average apparent within-herd prevalence of BVDV was 31.8%, ranging from 0% to 100%, and for BoHV-1 it was 62.4% ranging from 0% to 100%. Figures 3 and 4 show the kriging estimate of the apparent within-herd prevalences for BVDV and BoHV-1, respectively.

**Table 1:** Census data of the cattle population in the State of Paraíba, Northeastern Brazil, according to sampling stratum, and herd-level prevalence for BVDV and BoHV-1

Sampling stratum	No. of herds	Tested	BVDV			BoHV-1		
			Positive	Prevalence	95% CI	Positive	Prevalence	95% CI
Sertão	24,356	159	118	74.2	[66.8–80.4]	145	91.2	[85.6–94.7]
Borborema	11,603	160	79	49.4	[41.7–57.1]	130	81.3	[74.4–86.6]
Agreste/Zona da Mata	18,398	159	102	64.2	[56.4–71.3]	139	87.4	[81.3–91.8]
Stateof Paraíba	54,357	478	299	65.5	[61.1–69.7]	414	87.8	[84.5–90.5]

**Fig. 5:**
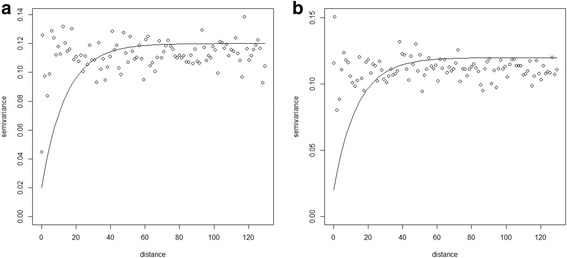
Sample semivariograms with fitted model (solid line) used to generate kriging surface of predicted within-herd prevalence of BVDV (**a**) and BoHV-1 (**b**)

Sample semivariograms with fitted exponential model are shown in Fig. 5. For BVDV, parameters were: nugget = 0.02, partial sill = 0.10, and range = 13.9 km; and for BoHV-1, parameters were: nugget = 0.01, partial sill = 0.10, and range = 13.0 km. The predicted prevalence was highest (0.42–0.75) for BVDV (Fig. 3a) in the west, north and eastern part of Sertão and in the central and eastern part of Agreste/Zona da Mata. For BoHV-1 (Fig. 4a), the highest predicted prevalence (0.74–0.97) was in some local areas across Sertão and thoughout the eastern part of Agreste/Zona da Mata. The prediction variance reflects the location of tested herds: low values in the western part of Sertão and north, central and southern part of Agreste/ Zona da Mata for BVDV (Fig. 3b) and north and central part of Agreste/Zona da Mata for BoHV-1 (Fig. 4b), where herd density was high.

**Fig. 6:**
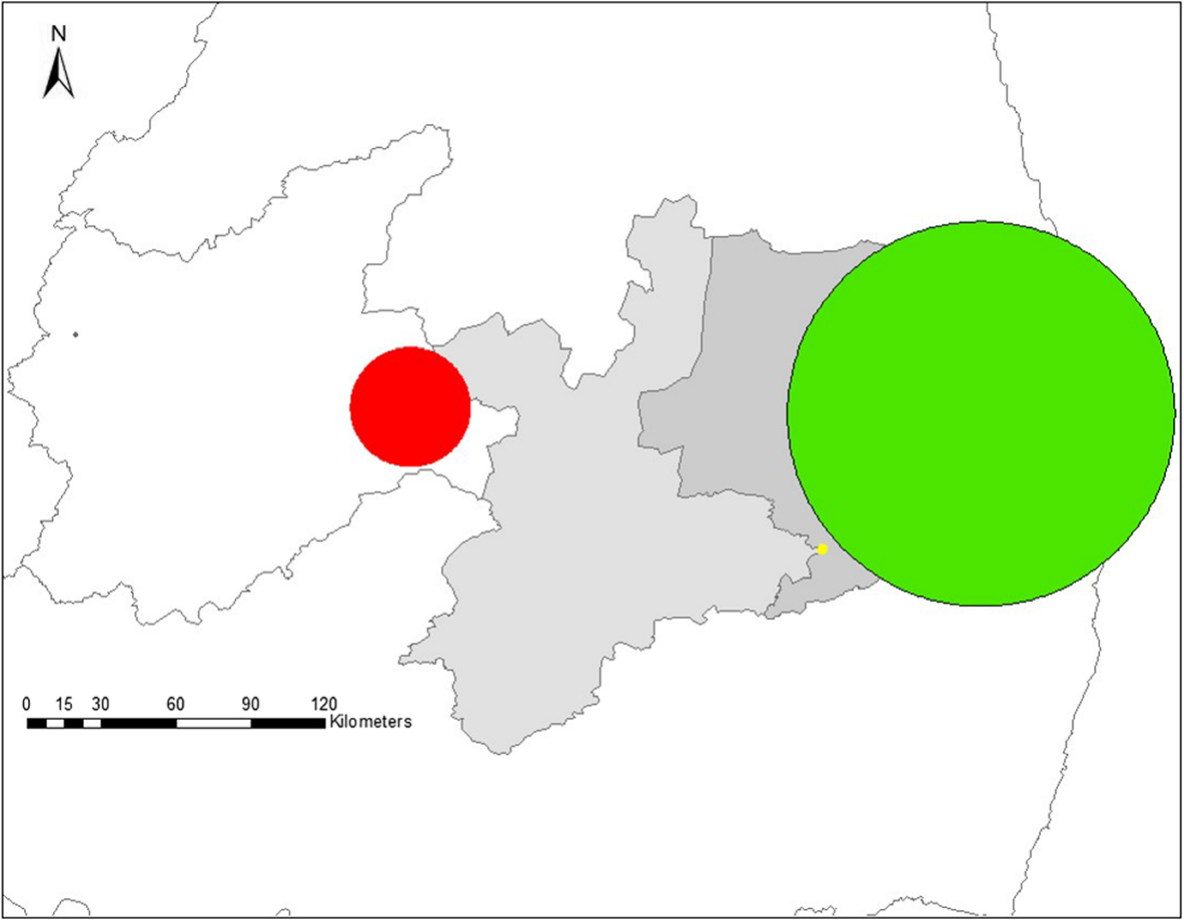
Significant clusters of herds with a high apparent within-herd prevalence of BVDV (primary cluster: red, secondary clusters: yellow) and BoHV-1 (green)

The Cuzick–Edwards’ test identified statistically significant (*P* <  0.05) spatial clustering of BVDV and BoHV-1 cases at *k* = 18 and *k* = 7 neighborhood levels, respectively. Using Bernoulli model, six significant clusters were detected for BVDV and one significant cluster for BoHV-1 (Table 2, Fig. 6). There was no spatial overlap among clusters. For BVDV, a primary cluster covered the eastern Sertão region, with 11 herds, radius of 24.10 km and the risk for infection was 2.21 (relative risk (RR) = 2.21; *P* <  0.001) times higher in herds located inside cluster than in those located elsewhere. The others significant BVDV clusters were smaller, involving one or two herds in Agreste/Zona da Mata region, near the border of the Borborema mesoregion, however, with a high relative risk. The BoHV-1 cluster covered the Agreste/Zona da Mata region with a radius of 77.17 km and a relative risk of 1.27 (*P* < 0.001), with 103 cases.

**Table 2:** Statistically significant clusters of herds with a high within-herd prevalence of BVDV and BoHV-1 in the state of Paraíba, Northeastern Brazil

Radius (km)	No. of herds in cluster	No. of cases in cluster		RR^a^	*p*-value
		Observed	Expected		
*BVDV*					
24.10^b^	11	55	25.87	2.21	< 0.001
2.11	2	8	2.59	3.11	0.045
0	1	13	4.53	2.90	0.004
0	1	10	3.23	3.12	0.005
0	1	10	3.23	3.12	0.005
0	1	10	3.23	3.12	0.005
*BoHV-1*					
77.17	103	359	297.39	1.27	< 0.001

^a^Relative risk

^b^Primary cluster

## Discussion

In Brazil, prevalence rates of BVDV range from 22.2% to 85.4% [18, 37, 38], and seropositivity frequencies of BoHV-1 between 18% and 90% were frequently observed in non-vaccinated herds throughout the different geographical areas of Brazil [39–41]. In the Northeastern Brazil, seroprevalences for BVDV infection of 72.6% [42] and 51.1% in family farms [43] in Pernambuco state, and for BoHV-1 infection of 79.5% [44] also in Pernambuco state, 62.67% [45] in Sertão of Paraíba and 63.23% [46] in Maranhão state have been reported. The herd-level seroprevalences for BVDV (65.5%) and BoHV-1 (87.8%) estimated for Paraíba state in this survey indicate that these viruses are present in most herds of the state.

In this work, six significant clusters were detected for BVDV infection and one significant cluster for BoHV-1. Similar results were found by kriging and SatScan for identification of risk areas (location of areas with high apparent within-herd prevalence) for BVDV and BoHV-1 infections: the western part of Sertão for BVDV and the western part of Agreste/Zona da Mata for BoHV-1.

The primary cluster for BVDV was detected in the Sertão region, with a radius of 24.10 km, and covering its eastern region. The Sertão was the stratum of higher prevalence for BVDV and is bordered on the north by Rio Grande do Norte state, west by Ceará state, and south by Pernambuco state. Border regions are always associated as risk factors, since in these regions there are large circulations of animals due to intense trade and often without knowledge of the health status of animals [47–49]. This circumstance corroborates what happens in the Sertão, where most of the properties are familiar or subsistence, predominating the mixed exploration, semi-confined farming, low technification and no veterinary assistance [50], increasing the risk in the herds of these properties.

Furthermore, recently BVDV was isolated in a herd in Pombal, a municipality of the Sertão mesoregion [51]. Knowing that PI animals are the main sources of infection of BVDV, and animal purching and pasture rental were identified as risk factors associated with herd-level seroprevalence for BVDV infection in the State of Paraíba [52], it is important to consider that the cluster for BVDV may have PI herds. Ersbøll et al. [53] in a study on the dynamics of BVDV infection among neighboring herds in Denmark found that the presence of PI-herds increased the risk of a herd becoming infected (PI-herd), showing *odds ratios* (OR) of 1.37, 1.40 and 1.70 for 1, 2, and ≥3 PI-herds in the neighborhood, respectively, as well as the risk of becoming a PI-herd was negatively associated with the mean distance from the neighboring herds (OR = 0.7 for an increase of 1 km), concluding that the occurrence of PI-herd in a certain area has influence on the risk of a neighbor herd becoming infected.

In Brazil, Hein et al. [54] found two BVDV clusters of dairy herds in the Arroio do Meio region, Rio Grande do Sul state, a primary (*P* = 0.391) and a secondary (*P* = 0.773) by scanning analysis, but both without statistical significance. According to the author, the occurrence of no association may have been due to the scan test limitation or because transmission of the virus via aerosols or other vehicles is not an effective way to disseminate. Kirchgessner et al. [55] in an exploratory cluster analysis identified clusters in different locations for domestic livestock and white-tailed deer in New York state, United States, suggesting that BVDV is maintained independently in domestic livestock herds in the western part of the state and in the white-tailed deer population in the northwestern part. According to the authors, the spatial point pattern analyses provide information necessary for the epidemiological risk assessment that should precede the development of any regional BVDV management plan.

The significant BoHV-1 cluster (*P* < 0.001) showed a radius of 77.17 km with 103 herds whose 359 animals were positive when it was expected 297 positive animals, and involved a large part of the Agreste/Zona da Mata stratum, which has a high herd-level prevalence (87.4%). Despite the seroprevalence is high throughout the state, the presence of a significant BoHV-1 cluster requires epidemiological interpretation of its spatial distribution and, consequently, the decisions based on that evidence [56]. The mesoregions of Agreste and Zona da Mata had the highest rainfall in the state and a bovine livestock characterized by dairy farms and predominant confined rearing [50]. Miranda et al. [57] detected BoHV-1 clusters in southeastern Brazil and observed that disease clusters may occur either because herds share common risk factors or via transmission between herds through the movement of infected animals. The high prevalence of BoHV-1 in the Agreste/Zona da Mata stratum and the presence of cluster suggest that the relationship between the cases and the spread of the virus is due to the proximity of the animals in the herd [20]. BoHV-1 clusters were detected in the state of Rio Grande do Sul, in which the most significant (*P* = 0.00027) had a radius of 122.33 km, with 173 properties, 109 positive animals, and RR of 1.31, showing a moderate presence of BoHV-1 infection in the southern herds [20].

In addition to the presence of PI animals for BVDV infection and purchasing untested cattle, other environmental or management factors may be influencing the intra and inter herd transmission for both infections in the state of Paraíba, mainly in high-risk areas. Such factors could be the lack of biosafety procedures in herds, extensive breeding, mixing cattle of different ages and from multiple sources, communal grazing and absence of vaccination.

In the risk mapping described in this study, there is a significant relation between the spatial effect and the development of the diseases, indicating that there are some important predisposing factors in the occurrence of infections studied. The herds of Paraíba and neighbor’s states might be sharing common risk factors or transmission routes among herds through the movement of infected animals, animal purchasing without the knowledge of the health status of animals, pasture rental, or absence of permanent veterinary assistance.

There may be other variables not considered in this work that can determine the spatial distribution pattern for both BVDV and BoHV-1 infections in a particular area. These variables may be potential risk factors that influence a higher risk of disease in areas of greater spatial effect, therefore, further works should be considered.

## Conclusions

The spatial analysis enabled the identification of spatial clustering of risk for BVDV and BoHV-1 in the state of Paraíba. The clusters detected contemplated different areas of the state, with BVDV cluster located in the Sertão stratum and BoHV-1 in Agreste/Zona da Mata stratum. Through the risk mapping, it was possible to identify the areas in which the risk is significantly elevated, coinciding with areas where there are borders with other states and in which there is a high movement of animals. Clusters detection analysis enabled a better view of the occurrence of the BoHV-1 and BVDV and its result indicates the highest risk areas in the state of Paraíba, facilitating control strategies for specific actions and directed to those areas.

## Abbreviations

BoHV-1: Bovine Herpesvirus type 1
BVDV: Bovine Viral Diarrhea Virus
GPS: global positioning system
OR: odds ratio
PI: persistently infected
RR: Risk Relative
SEDAP: Agricultural and Livestock Defense Service of the State of Paraíba
TCID_50_: 50% Tissue culture Infective Dose
